# Integrated Omics Strategy Reveals Cyclic Lipopeptides Empedopeptins from *Massilia* sp. YMA4 and Their Biosynthetic Pathway

**DOI:** 10.3390/md19040209

**Published:** 2021-04-09

**Authors:** Shang-Tse Ho, Ying-Ning Ho, Chih Lin, Wei-Chen Hsu, Han-Jung Lee, Chia-Chi Peng, Han-Tan Cheng, Yu-Liang Yang

**Affiliations:** 1 Agricultural Biotechnology Research Center, Academia Sinica, Taipei 11529, Taiwan; stho@mail.ncyu.edu.tw (S.-T.H.); chih@macrogen-europe.com (C.L.); ny450130@gate.sinica.edu.tw (W.-C.H.); millettaipei@tamu.edu (H.-J.L.); Chia-Chi.Peng@hki-jena.de (C.-C.P.); r04623008@ntu.edu.tw (H.-T.C.); 2 Department of Wood Based Materials and Design, College of Agriculture, National Chiayi University, Chiayi 60004, Taiwan; 3 Institute of Marine Biology, College of Life Science, National Taiwan Ocean University, Keelung 20224, Taiwan; ynho@mail.ntou.edu.tw; 4 Center of Excellence for the Oceans, National Taiwan Ocean University, Keelung 20224, Taiwan

**Keywords:** *Massilia*, lipopeptides, biosynthesis, genome mining, metabolomics

## Abstract

Empedopeptins—eight amino acid cyclic lipopeptides—are calcium-dependent antibiotics that act against Gram-positive bacteria such as *Staphylococcus aureus* by inhibiting cell wall biosynthesis. However, to date, the biosynthetic mechanism of the empedopeptins has not been well identified. Through comparative genomics and metabolomics analysis, we identified empedopeptin and its new analogs from a marine bacterium, *Massilia* sp. YMA4. We then unveiled the empedopeptin biosynthetic gene cluster. The core nonribosomal peptide gene null-mutant strains (Δ*empC*, Δ*empD*, and Δ*empE*) could not produce empedopeptin, while dioxygenase gene null-mutant strains (Δ*empA* and Δ*empB*) produced several unique empedopeptin analogs. However, the antibiotic activity of Δ*empA* and Δ*empB* was significantly reduced compared with the wild-type, demonstrating that the hydroxylated amino acid residues of empedopeptin and its analogs are important to their antibiotic activity. Furthermore, we found seven bacterial strains that could produce empedopeptin-like cyclic lipopeptides using a genome mining approach. In summary, this study demonstrated that an integrated omics strategy can facilitate the discovery of potential bioactive metabolites from microbial sources without further isolation and purification.

## 1. Introduction

The production of secondary metabolites is regulated by several genes that are responsible for modifying their chemical structure, transporting substrates and products, and other specific regulatory functions. These genes, named secondary metabolite biosynthesis gene clusters (BGCs), are contiguously aligned in the genome [1]. Advances in computational science and bioinformatic analysis now provide the possibility of dissecting secondary metabolite biosynthetic pathways from microorganisms [2]. Comparative genomics analysis and BGC mining have been applied to the natural product studies to discover new sources of bioactive natural products [3]. In addition, several mass spectrometry based metabolomics analysis approaches, such as Global Natural Products Social Molecular Networking (GNPS), have been utilized to monitor the production of specific molecules [4]. Molecular networking groups similar molecules as a cluster by comparing vector correlations between tandem mass fragment ions of molecules [5]. Thus, integrated BGC mining and molecular networking analysis are well suited for dereplication and identification of specific metabolites efficiently and even proposes new analog structures.

The biodiversity of marine microorganisms is exceptionally high and contributes to the vast chemical diversity seen among their related metabolites [6]. The chemical diversity of metabolites might play vital roles in their bioactivities. One of the significant applications of natural products is antibiotics. As reported previously, approximately 80 natural product-related antibiotics were approved by the FDA from 1981 to 2014 [7]. However, as is now well-known, existing antibiotics cannot effectively treat resistant strains of bacterial infections. Therefore, seeking new antibiotics from natural products is a priority. *Staphylococcus aureus* is a commensal bacterium in humans that colonizes in approximately 30% of the human population. It is also a major human pathogen and the leading cause of bacteremia, infective endocarditis, and other infections in the clinic [8]. Therefore, screening natural resources for effective agents against *S. aureus* infection is a potentially worthwhile challenge.

In this study, we first applied comparative genomics analysis and GNPS to reveal the biosynthetic gene cluster and new analogs of empedopeptin derived from the marine-derived bacterium *Massilia* sp. YMA4. We further confirmed empedopeptins as the main antibiotic utilized by *Massilia* sp. YMA4 against *S. aureus*, using an insertional mutation approach of the biosynthetic genes. We then utilized genome mining to explore new bacterial sources of empedopeptins and related cyclic lipopeptides. Such an integrated approach holds promise for discovering new natural sources of antibiotics and their production.

## 2. Results and Discussion

### 2.1. Antibiotic Activity of Massilia *sp.* YMA4

**Figure 1:**
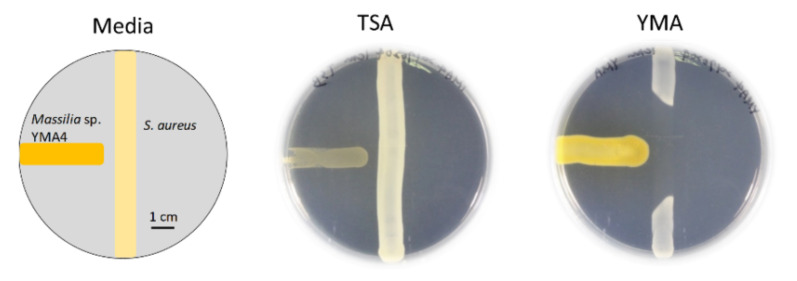
Antagonistic assays of *Massilia* sp. YMA4 versus *S. aureus* in yeast malt agar (YMA) and tryptone soy agar (TSA) culture conditions.

*Massilia*, a Gram-negative bacteria genus, was first found in clinical blood samples in 1998 [9] and was subsequently isolated from various environmental samples [10]. Some strains of the *Massilia* genus, such as *Massilia* sp. BS-1 and *Massilia* sp. NR 4-1 were reported to be antibiotic producers [11,12]. However, knowledge about the potential natural antibiotics related to nonribosomal peptides in the *Massilia* remains scarce. For the screening of new antibiotics, a strain named *Massilia* sp. YMA4 was isolated from a sediment sample collected in the open ocean off Lamay island in Taiwan. The antagonist assay revealed that *Massilia* sp. YMA4 effectively inhibited the growth of *S. aureus* ATCC 29213 only in yeast malt agar (YMA), but not in tryptone soy agar (TSA) culture conditions (Figure 1). To further understand the phylogenetic relationships of *Massilia* sp. YMA4 to other sequenced *Massilia* strains, the whole genome sequences of 69 available *Massilia* strains deposited in the NCBI database (2020/02) were collected and analyzed using the maximum likelihood method. *Massilia* sp. YMA4 was clustered with *M. armeniaca* ZMN-3 (Figure S1). Interestingly, *Massilia* sp. YMA4 and *M. armeniaca* ZMN-3 were close to *Empedobacter halobium* ATCC 31962 according to BLAST analysis using partial 16S rRNA gene [13]. *E. halobium* ATCC 31962 has also been reported to have antimicrobial potential against Gram-positive bacteria [14].

### 2.2. The Biosynthetic Potential of Secondary Metabolites of the Massilia Strains and the Discovery of the Empedopeptin Biosynthesis Gene Cluster

**Figure 2:**
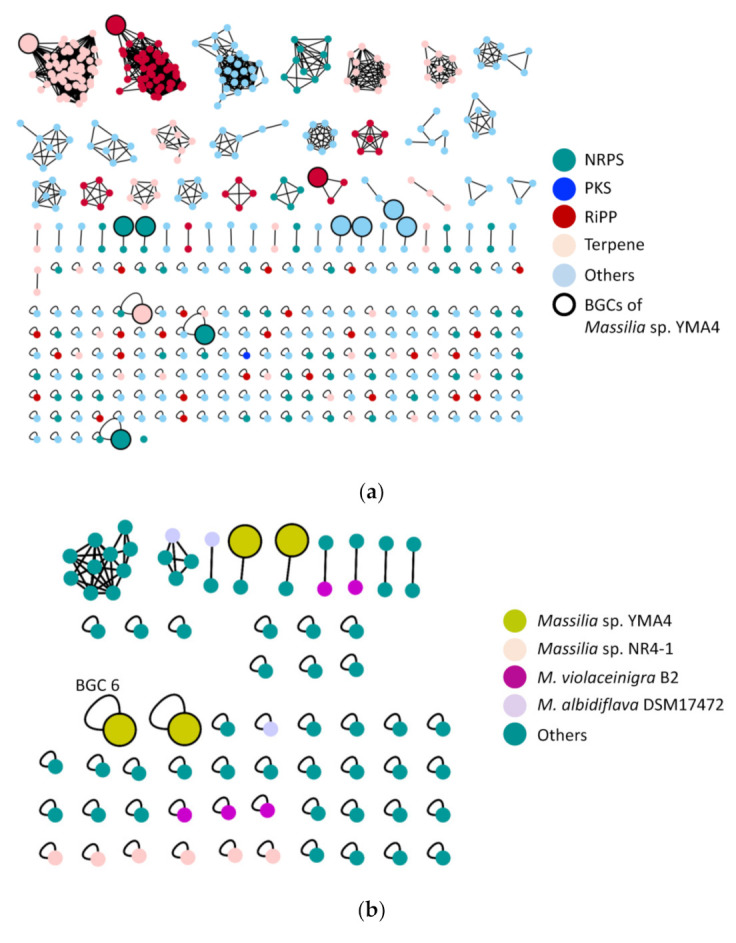
Sequence similarity network of biosynthetic gene clusters (BGCs) from 72 *Massilia* strains analyzed by antiSMASH. (**a**) Networks of all the predicted secondary metabolite BGCs. (**b**) Networks of nonribosomal peptides BGCs. Each node represents one BGC, and the similar BGCs were linked together as one cluster. The single-node indicates unique BGC in the network. All networks were generated by BiG-SCAPE and illustrated with Cytoscape. BGC 6 is the empedopeptin BGC. NRPS: non-ribosomal peptide synthetase; PKS: polyketide synthase; RiPP: ribosomally synthesized and post-translationally modified peptides.

A computational analysis of the secondary metabolite biosynthetic capacity of 72 *Massilia* strains was conducted using antiSMASH 5.1, MiBIG, and BiG-SCAPE [15,16]. The results revealed a high diversity of BGCs in the *Massilia* (Figure 2a). In 72 *Massilia* strains, 490 BGCs were identified, and the homologous BGCs were further grouped into 233 gene cluster families (GCF). Interestingly, 75 nonribosomal peptide BGCs were predicted (NRPS BGC). However, only four *Massilia* strains, *Massilia* sp. NR 4-1 (6 NRPS BGCs), *M. violaceinigra* B2 (5 NRPS BGCs), *Massilia* sp. YMA4 (4 NRPS BGCs), and *M. albidiflava* DSM17472 (3 NRPS BGCs) contained more than three NRPS BGCs (Figure 2b).

**Figure 3:**
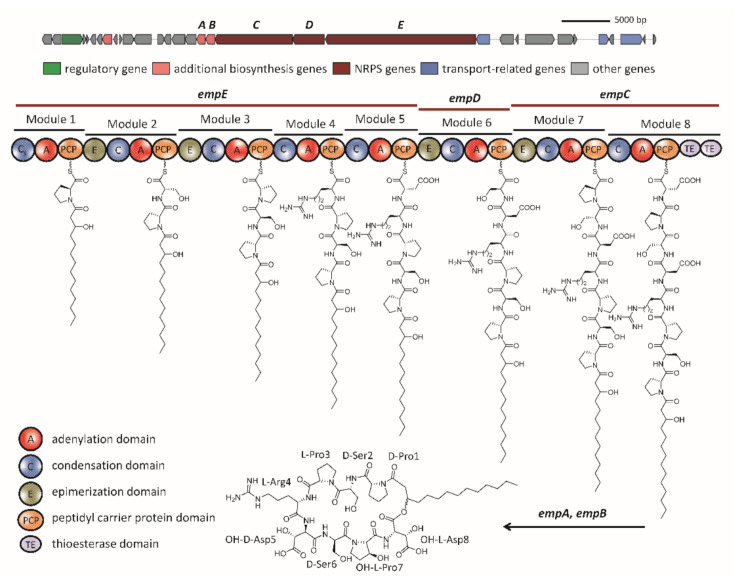
In silico analysis of empedopeptin biosynthetic gene cluster and flanking regions of *Massilia* sp. YMA4. The empedopeptin biosynthetic gene cluster was predicted by using antiSMASH5.1. Scale bar = 5 kb.

Twelve putative BGCs were predicted in the *Massilia* sp. YMA4 genome, including four NRPS BGCs (Table S1). Among them, BGC 6 was comprised of three NRPS-related genes, *empC* (two modules, 8.2 kb), *empD* (one module, 3.3 kb), and *empE* (five modules, 15.9 kb); and two additional genes, *empA* (dioxygenase, 0.9 kb) and *empB* (dioxygenase, 0.9 kb) (Figure 3). The modules of *empC*, *empD*, and *empE* genes were comprised of one condensation (C), one adenylation (A), and one peptidyl carrier protein (PCP) domain. In addition, two thioesterase (TE) domains were located in *empC* (Figure 3).

The A domain in each module was further analyzed using antiSMASH 5.1. and NRPSPredictor2 [15,17], which suggests that the amino acid sequence of the BGC 6 product is: Pro1-Ser2-Pro3-Arg4-Asp5-Ser6-Pro7-Asp8. Additionally, a lipo-initiation (CStarter) domain in module 1 of *empE* was revealed by antiSMASH 5.1 and NaPDos [18]. These results demonstrated that this domain is similar to the CStarter of syringomycin, indicating that this domain can catalyze the acylation of the first amino acid [19]. The combination of the polar peptide and fatty acid tail is a key feature of cyclic lipopeptides that is responsible for their amphiphilic properties [20]. The amphiphilic properties of lipopeptides have great potential for pharmaceutical use and can also deliver drugs by forming active pharmaceutical ingredients as liposomes [21]. The remaining C domains were further analyzed. Among these C domains, modules 2, 3, 6, and 7 were predicted as combined C/E (epimerization) domains, while the other C domains are the conventional ^L^C_L_ domains. The E domain contributes to the stereochemistry of amino acid residues in the NRPS assembly line. It changes the l form amino acid to the d form amino acid (^L^C_D_) [22]. Therefore, Pro1, Ser2, Asp5, and Ser6 are in the d configuration, while Pro3, Arg4, Pro7, and Asp8 are in the l configuration. Interestingly, most NRPS BGCs have one TE domain in the last module, but our results revealed two TE domains in BGC 6. The two TE domains in the last module have also been found in some cyclic lipopeptides BGCs, such as orfamide A, viscosin, and massetolide produced by *P. fluorescens* strains [23]. There are two types of TE domains, type I and II, which exhibit different functions. The type I TE domain can catalyze intramolecular cyclization, while the type II TE domain functions and supports the NRPS assembly line [24]. Taken together, the results of the bioinformatic analysis indicated that the structure produced by the BGC 6 is 3OH-FA-d-Pro1/d-Ser2/l-Pro3/l-Arg4/d-Asp5/d-Ser6/l-Pro7/l-Asp8, which is the same as the core structure of empedopeptin (Figure 3).

### 2.3. Molecular Networking of Empedopeptin and Its Analogs from Massilia *sp.* YMA4 Wild-Type and Mutant Strains

**Figure 4:**
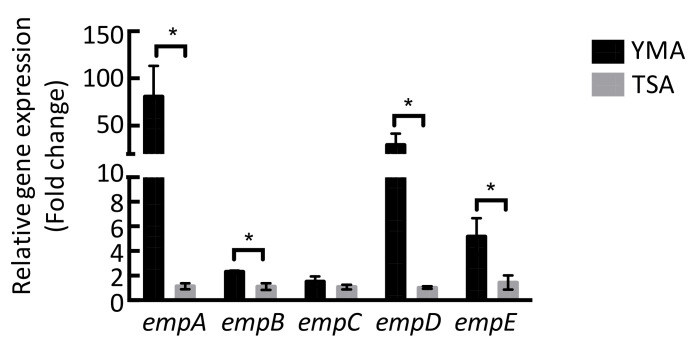
Relative gene expression of empedopeptin biosynthetic-related genes in different culture conditions (mean ± SD, *n* = 5). The statistical analysis was conducted using the student’s t-test. * *p* < 0.05.

We evaluated the potential empedopeptin biosynthetic-related gene expression under various culture conditions (Figure 4). The results were consistent with the antagonistic assay results, suggesting that the TSA medium could not activate the empedopeptin biosynthetic-related gene expression and thus was unable to inhibit *S. aureus* ATCC 29213 growth (Figure 1).

We, therefore, extracted the metabolites from the *Massilia* sp. YMA4 for LC-MS/MS analysis. One significant signal of empedopeptin at *m/z* 1126.5630 was observed in the extract, suggesting that this compound might act as the main contributor to the antibiotic activity of *Massilia* sp. YMA4 to *S. aureus* ATCC 29213 (Figure S2). The structure of empedopeptin was further confirmed by LC-MS/MS that a base peak at *m/z* 1126.5630 [M + H]^+^, consistent with a molecular formula of C_49_H_79_N_11_O_19_ and an index of hydrogen deficiency (IHD) of 16. The fragments of b/y ions of empedopeptin are listed in Figure S2. Fragments of b ions in order were, 977.5298 (b7), 864.4842 (b6), 777.4520 (b5), 646.4296 (b4), and 393.2738 (b2), while the fragments of y ion were 918.3830 (y8), 821.3257 (y7), 734.2978 (y6), and 637.2446 (y5). Additionally, we found that the Asp5, Ser7, and Asp8 contain a hydroxyl group.

**Figure 5:**
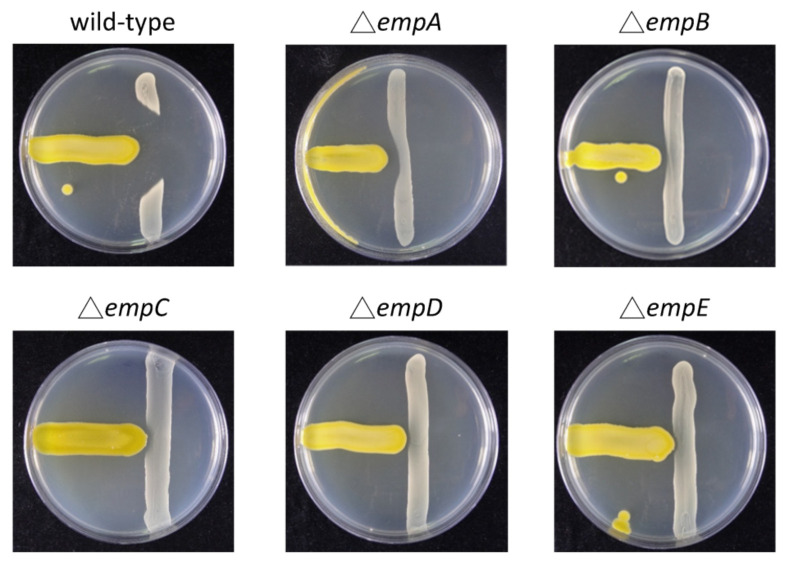
Antagonistic assays of the wild-type and core empedopeptin biosynthetic gene null-mutant strains (Δ*empA-E*) of *Massilia* sp. YMA4 versus *S. aureus* on YMA.

**Figure 6:**
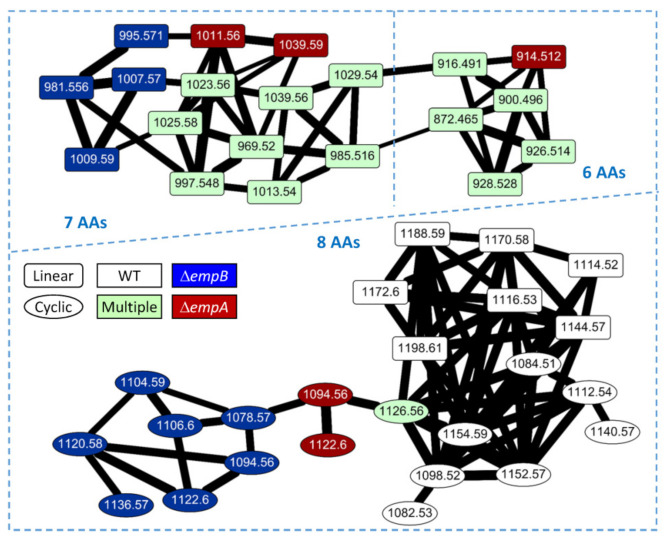
Molecular networking of empedopeptin analogs from *Massilia* sp. YMA4 wild-type (WT) and null-mutant strains. 6 AAs: 6 amino acids lipopeptides; 7 AAs: 7 amino acids lipopeptides; and 8 AAs: 8 amino acids lipopeptides. Each node represents one lipopeptide tandem mass spectrum, and the width of the edge indicates the tandem mass spectral similarity of neighboring nodes. The shape of the node represents linear or cyclic lipopeptide. The color of the node represents the source of the lipopeptide. Multiple represents the analog was detected from more than one strain.

To further understand the biosynthetic pathway of empedopeptin, we constructed the *empA*, *empB*, *empC*, *empD*, and *empE* null-mutant strains. The mutant strains, Δ*empC*, Δ*empD*, and Δ*empE*, could not inhibit *S. aureus* ATCC 29213 growth, and Δ*empA* and ΔempB strains showed significantly reduced inhibition effects compared to wild-type (Figure 5). We then conducted a comparative metabolomic analysis (molecular networking) of the wild-type and null-mutant strains to elucidate the structures and biosynthetic pathways of empedopeptin and its analogs. We identified 44 empedopeptin analogs, including 17 cyclic lipopeptides and 27 linear lipopeptides (Figure 6). As shown in Figure 6, we observed the empedopeptin analogs only from wild-type, Δ*empA*, and Δ*empB* strains (dioxygenase genes), suggesting that the Δ*empC*, Δ*empD*, and Δ*empE* strains (core NRPS genes) were unable to produce empedopeptins. Additionally, we found several unique analogs that only appear in the molecular networking analysis of Δ*empA* and Δ*empB* strains. In the lipopeptides containing eight amino acids, *m/z* 1094.56 and 1122.6 were found in both Δ*empA* and Δ*empB* strains, while *m/z* 1078.57, 1104.59, 1106.6, 1120.58, and 1136.57 were observed only in the Δ*empB* strain. Comparing the structure of *m/z* 1094.56 and 1122.6 from Δ*empA* and Δ*empB* strains showed that they comprise the identical amino acid sequences as empedopeptin, but have hydroxyl modification in different amino acids and different chain lengths of 3-hydroxyl fatty acid as well. Asp5, Pro7, and Asp8 residues were all hydroxylated in empedopeptin. We found cyclic lipopeptides containing OH-Asp5 together with unmodified Pro7 and Asp8 from Δ*empA* strain, while cyclic lipopeptides with OH-Asp8 and even no hydroxylated residues were only identified from Δ*empB* strain (Table S2). These results demonstrated that the dioxygenase genes, *empA* and *empB*, located at the end of the empedopeptin biosynthetic assembly line, functioned in hydroxylation and played an important role in the bioactivity of cyclic lipopeptides. In addition to the cyclic lipopeptides, we also identified several linear lipopeptides, which might be the biosynthetic intermediates of empedopeptins (Figure 6). The structure information of these empedopeptin analogs is summarized in Tables S2 and S3.

### 2.4. Genome Mining for Discovering Other Empedopeptin-Like Compound Producing Bacteria

**Figure 7:**
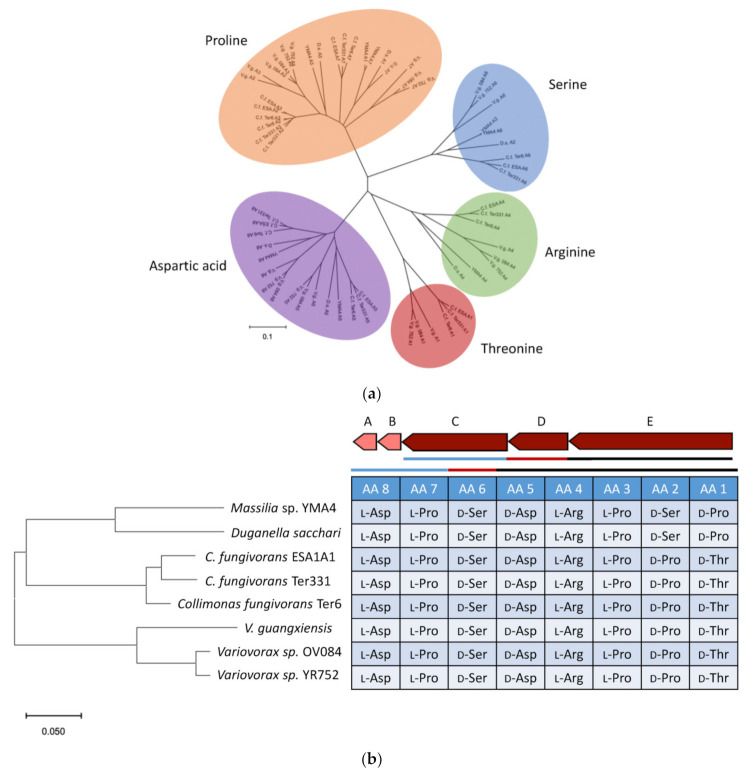
Phylogenetic analyses of adenylation (A) domains and biosynthetic gene clusters (BGCs) of empedopeptin-like cyclic lipopeptides. (**a**) Phylogenetic analysis of A domains in BGCs of empedopeptin-like cyclic lipopeptides. (**b**) Phylogenetic analysis of BGCs of empedopeptin-like cyclic lipopeptides and their proposed amino acid sequences. AA1-AA5 from gene E; AA6 from gene D; AA7-AA8 from gene C.

Through the MultiGeneBlast analysis, we found that several microorganisms may produce cyclic lipopeptides similar to empedopeptins because similar NRPS BGCs were observed. The adenylation domain plays a role in selecting and recruiting specific amino acids in the NRPS biosynthetic pathway [25]. Therefore, to further propose the structures of cyclic lipopeptides, all the adenylation domains in the empedopeptin-related BGCs were analyzed phylogenetically (Figure 7a). Five types of amino acids, arginine, aspartic acid, proline, serine, and threonine, were involved in those BGCs. As shown in Figure 7b, *Duganella sacchari* has the same NRPS BGC for producing empedopeptin-like compounds. However, *Collimonas* and *Variovorax* strains were proposed to be the producers of tripropeptin-like compounds [26].

In the present study, we aimed to explore the antibiotic substances from the marine bacterium *Massilia* sp. YMA4 by integrating comparative genomics and metabolomics analysis. Our results revealed that the primary active substance of *Massilia* sp. YMA4 is a nonribosomal peptide, empedopeptin. We found that *Massilia* sp. YMA4 only inhibited *S. aureus* ATCC 29213 under certain culture conditions, suggesting that the empedopeptin biosynthetic gene cluster might be a silent gene cluster activated by changing culture conditions. This result was consistent with a previous study, demonstrating that microbial secondary metabolite synthesis is influenced by changing the type and concentration of nutrients in the culture media [27].

Empedopeptin is a calcium-dependent antibiotic that acts against Gram-positive bacteria by inhibiting cell wall biosynthesis [28]. Certain calcium-dependent antibiotics contain a conserved Asp-X-Asp-Gly motif that is thought to facilitate calcium binding [29]. This study confirmed that two dioxygenase genes, *empA* and *empB*, contributed to the hydroxylation of the amino acid residues in empedopeptin. The Δ*empA* and Δ*empB* strains showed significantly reduced inhibition effects compared to the wild-type, which implies the hydroxylated amino acid residues are important to antibiotic activity. We speculated that the two dioxygenases worked synergistically to hydroxylate the amino acid residues of empedopeptin. However, it is challenging to validate the hydroxylation selectivity of these two dioxygenases to specific amino acid residues or peptide sequences.

Some bacteria were found to possess cyclic lipopeptide BGCs that are similar to empedopeptin. However, the amino acid residues of those cyclic lipopeptides were different. As shown in Figure 7b, the first and second residues of cyclic lipopeptides of *Collimonas* and *Variovorax* strains encode threonine and proline, respectively, while the same positions in *Massilia* sp. YMA4 and *D. sacchari* encode proline and serine, respectively. Surprisingly, we did not find the empedopeptin-related BGC from empedopeptin-producing bacteria *E. haloabium* ATCC 31962 through MultiGeneBlast analysis [13,30]. Therefore, we speculate that the whole genome of *E. haloabium* ATCC 31962 might not be well sequenced.

## 3. Materials and Methods

### 3.1. Bacterial Culture Conditions and Antagonistic Assay

*Massilia* sp. YMA4 was isolated from a marine sediment core collected by research vessel Ocean Researcher No. 3, offshore of Lamay island, Pingtung County, Taiwan, on 9 November, 2013 (OR3-1727). A voucher specimen (BCRC 81003) was deposited in the Bioresource Collection and Research Center (BCRC), Food Industry Research and Development Institute, Hsinchu, Taiwan. The 16S rRNA gene sequencing (Accession: KX444135) was affiliated with the genus *Massilia*. *Massilia* sp. YMA4 was cultivated in yeast malt broth (YMB) consisting of 3 g/L yeast extract; 3 g/L malt extract; 10 g/L dextrose; 5 g/L peptone at 30 °C, with orbital shaking (120 rpm) for 24 h. The barcoding gene, 16S rRNA sequence, was used to identify the strain as a *Massilia* sp. *S. aureus* ATCC 29213 was purchased from BCRC. For the antagonistic assay, *Massilia* sp. YMA4 was grown on tryptone soy broth (TSB) or YMB overnight at 30 °C. When the O.D. value of bacterial cultures reached 2.0, the bacteria were then inoculated to TSA and YMA plates at 30 °C. After two days of incubation, *S. aureus* ATCC 29213 (grown in LB, O.D. = 2.0) were then transferred to the same agar plates to perform the confrontation test at 30 °C.

### 3.2. DNA Sample Preparation, Whole-Genome Sequencing, Assembly, and Annotation

Genomic DNA of *Massilia* sp. YMA4 was extracted from the cultures grown at 30 °C in YMB medium using a genomic DNA purification kit (QIAgen, Hilden, Germany) following the manufacturer’s instructions. The genomic DNA (total 20 μg) was sequenced by the PacBio RS II system (Pacific Biosciences, Menlo Park, CA, USA). A 10-kb SMRTbell library was generated using a DNA Template Prep Kit 2.0 (10–20 Kb; Pacific Biosciences), following the manufacturer’s instructions. Sequencing was performed with a PacBio RS II sequencer using one SMRT cell and P6-C4 chemistry at 360 min movie length (Pacific Biosciences, Menlo Park, CA, USA). Single-molecule real-time reads (159,840 filtered subreads and a mean length of 11,850 bp) were de novo assembled using the Hierarchical Genome Assembly Process (HGAP) workflow in the SMRT analysis software version 3 (Pacific Biosciences Inc., Menlo Park, CA, USA) [31]. Genome annotation was performed via Rapid Annotations using Subsystems Technology (RAST) server version 2.0 and blast2go [32,33]. The whole-genome sequence of *Massilia* sp. YMA4 was uploaded to the Genbank database (GenBank assembly accession: GCA_003293715.1).

### 3.3. Genome Mining and Bioinformatic Analysis

The whole-genome sequence of *Massilia* sp. YMA4 was analyzed by antiSMASH 5.1 for the genome mining of possible secondary metabolite biosynthetic gene clusters [15]. A phylogenetic tree based on assembled genome sequencing data of genus *Massilia* from NCBI, including *Massilia* sp. YMA4 was performed using reference alignment-based phylogenetic builder (REALPHY) via bowtie2 [34]. The phylogeny was estimated on the “polymorphisms_move.phy” file produced by REALPHY via RAxML with the general time reversible (GTR) nucleotide substitution model and GAMMA model of rate heterogeneity [35]. Finally, RAxML was carried out with 1000 alternative runs on distinct starting trees (N) and a search for the best-scoring ML tree with 1000 replications. The phylogenetic tree was generated by using iTOL [36]. All BGCs associated with the genus *Massilia*, a total of 490 BGCs containing 233 gene cluster families, were downloaded and used for the sequence similarity network. The BIG-SCAPE-CORASON pipeline was utilized locally to analyze the 490 BGCs downloaded from the antiSMASH database (March 2019) [15,16]. The singleton parameter in the sequence similarity network was the BGCs with distances lower than the default cutoff distance of 0.3. Generated sequencing similarity network files separated by BiG-SCAPE class were combined for visualization using Cytoscape version 3.7.1 [37].

Each domain of empedopeptin BGC was predicted using antiSMASH 5.1 or NRPSpredictor2 [17]. The screening of empedopeptin-related biosynthetic genes from various microorganisms was performed using MultiGeneBlast [30]. For the MultiGeneBlast analysis, a local MultigeneBlast database was applied in this study, built with 101,223 bacterial genomic sequences from the GenBank assembly database of NCBI (download date: March 2019) and processed by MultigeneBlast under a Linux-based system. The sequences from *empA* to *empE* were the query template. The whole-genome sequences of *C. fungivorans* ESAIA1 (GenBank assembly accession: GCA_003610095.1), *C. fungivorans* Ter331 (GenBank assembly accession: GCA_000221045.1), *C. fungivorans* Ter6 (GenBank assembly accession: GCA_001584145.1), *D. sacchari* (GenBank assembly accession: GCA_900143065.1), *E. haloabium* ATCC 31962 (GenBank assembly accession: GCA_008011715.1), *V. guangxiensis* (GenBank assembly accession: GCA_003952165.1), *Variovorax* sp. OV084 (GenBank assembly accession: GCA_900111625.1), and *Variovorax* sp. YR752 (GenBank assembly accession: GCA_900215425.1) were downloaded from the GenBank database (NCBI). Phylogenic analysis sequences of empedopeptin-related biosynthetic genes of selected microbial strains were then analyzed to compare the similarity of empedopeptin-related biosynthetic genes between each strain (Table S4). The phylogenic analysis of empedopeptin-related genes was constructed with Molecular Evolutionary Genetics Analysis version X (MEGAX) [38]. Predicted BGCs among different genomes were analyzed using a modified Pfam domain similarity metric implemented in BigScape for the similarity network of the BGCs [39,40]. A cutoff of 0.75 was used for the analysis [40].

### 3.4. Construction of Empedopeptin Biosynthetic Gene-Null Mutant Strains

To make insertion mutations of empedopeptin biosynthetic genes *empA*, *empB*, *empC*, *empD*, and *empE* in *Massilia* sp. YMA4, pCM184-Δ*empA*, pCM184-Δ*empB*, pCM184-Δ*empC*, pCM184-Δ*empD*, and pCM184-Δ*empE* were constructed. The schematic diagram of the insertion mutant is shown in Figure S3. The pCM184 was purchased from Addgene (MA, USA). The regions for homologous recombination in *Massilia* sp. YMA4 were amplified by primer pairs, which are listed in Tables S5 and S6. The PCR products were digested with KpnI and SacI enzymes and cloned into the KpnI/SacI site of the digested pCM184, followed by transformation into the *E. coli* S17-1 λ pir. The *E. coli* S17-1 was applied to transform pCM184-Δ*empA*, pCM184-Δ*empB*, pCM184-Δ*empC*, pCM184-Δ*empD*, and pCM184-Δ*empE* individually into *Massilia* sp. YMA4 in this study. Both *Massilia* sp. YMA4 and *E. coli* S17-1 were cultured overnight in YMB at 30 °C and in LB at 37 °C, respectively. Both cultures were then washed by YMB twice, and the pellets were suspended by YMB again. The O.D.600 values of the donor (*E. coli* S17-1) and recipient cells (*Massilia* sp. YMA4) were 2.0, then the donor and recipient cells were mixed at 1:1 (*v*:*v*) ratio. The mixtures were spotted on YMA for overnight inoculation. The colonies were scraped up and spread on YMA with tetracycline (2 μg/mL) and kanamycin (50 μg/mL), and then the conjugants were picked and checked by PCR after 48 h. The conjugants could grow if the plasmid was integrated into the *Massilia* sp. YMA4 genomic DNA. The insertion sites were confirmed by PCR (Figure S4).

### 3.5. Quantitative Reverse Transcription PCR (qRT-PCR)

To evaluate *emp* biosynthetic gene expressions of *Massilia* sp. YMA4 on TSA and YMA culture conditions, the *empA*, *empB*, *empC*, *empD*, and *empE* were analyzed by qRT-PCR. Firstly, *Massilia* sp. YMA4 was grown on TSB or YMB overnight at 30 °C. When the O.D.600 values of bacterial cultures reached 2.0, the 100 μL of bacterial cultures were then spread on TSA and YMA plates at 30 °C. After two days, the bacterial cells on TSA and YMA were scratched and collected in 1.5-mL microcentrifuge tubes. Total RNA of bacterial cells was extracted using the TRIZOL (Ambion, Waltham, MA, USA) reagents following the manufacturer’s instructions. The resulting RNA samples were dissolved in 20 μL RNase-free water and then qualified using a NanoDrop 1000 Spectrophotometer (Thermo Fisher Scientific, Waltham, MA, USA). The cDNA samples were reverse transcribed from RNA templates using the ToolsQuant II Fast RT Kit (BioTools, New Taipei City, Taiwan) according to the manufacturer’s instructions. In brief, 8 μL of total RNA template (2 μg) was mixed with 2 μL of 5× gDNA Eraser and then incubated at 42 °C for 3 min to remove genomic DNA. The gDNA removal reaction mixtures were mixed with 1 μL of RT Enzyme Mix, 2 μL of RT Primer Mix, 2 μL of 10× Fast RT Buffer, and 5 μL RNase-free water and incubated at 42 °C for 15 min. Terminate the reactions at 95 °C for 3 min. The synthesized cDNA samples were stored at −20 °C until analyzed.

The primer sets used for the qRT-PCR were listed in Table S5. The qPCR reaction consisted of 10 μL of SYBR Green Master Mix (BioTools, New Taipei City, Taiwan), 0.4 μL of each of the forward and reverse primers (100 μM), 2 μL of cDNA template, and 7.2 μL of ddH_2_O for a total volume of 20 μL. The reactions were further amplified by the following thermocycling steps: 95 °C for 5 min; 39 cycles of 95 °C for 10 s (denaturation), 60 °C for 5 s (annealing), 72 °C for 30 s (extension), and then conducted melting curve analysis (95 °C for 15 s, 60 °C for 5 s, then increased 0.5 °C/s up to 95 °C). The RT-qPCR analysis was performed in CFX96™ Real-Time PCR Detection System with C1000™ thermal cycler (Bio-Rad, Hercules, CA, USA) with five replicates. The results were analyzed using the CFX Manager software, the delta CT (cycle of threshold) was detected to determine the relative expression level of genes. All target genes were further normalized to the bacterial 16S gene (Internal control).

### 3.6. Comparative Metabolomics and Molecular Networking Analysis

For comparative metabolomics, *Massilia* sp. YMA4 and mutants were cultured on YMA until colony formation. The single colony of *Massilia* sp. YMA4 was picked and transferred to 3 mL YMB for overnight incubation. For the mutants, the single colony was transferred to 3 mL YMB with 0.1% tetracycline. After overnight incubation, 0.5 mL of *Massilia* sp. YMA4 or mutant cultures were transferred to 500 mL YMB w/wo 500 μg tetracycline for three days. The resulting culture broth was extracted by EtOAc. The remaining broth was subsequently extracted with BuOH to obtain BuOH extracts. The BuOH extracts of each strain (10 mg/mL) were dissolved in MeOH. Therefore, 10 μL of extracts were injected and separated by a C18 column (ACQUITY UPLC BEH C18, 1.7 μm, 2.1 × 100 mm, Waters, Milford, MA, USA) with the following gradient: 0–8 min at 5–99.5% of B (A: ACN: H_2_O = 2: 98 plus 0.1% formic acid; B: ACN plus 0.1% formic acid), 8–10 min at 99.5% of B, 10–10.2 min at 99.5–5% of B, 10.2–12 min at 5% of B. Flow rate was set at 0.4 mL/min. The mass data were acquired using UPLC-ESIMS (Thermo Scientific Orbitrap Elite Mass Spectrometer), and the mass range was set up as *m*/*z* 100–1500. The mass data were acquired in profile mode for molecular networking analysis, positive mode ion detection between *m*/*z* 100–1500 with 30,000 resolution. The top four intense ions from each full mass scan were selected for collision-induced dissociation (CID) fragmentation. For CID, isolation width was 2 Da, and the selected ions were fragmented with normalized collision energy 30.0, activation Q 0.250, activation time 10.0, and 15,000 resolution. The mass data (.RAW files) from Xcalibur were converted to mzML file format using ProteoWizard software and subjected to GNPS (https://gnps.ucsd.edu/ProteoSAFe/static/gnps-splash.jsp, accessed on 20 March 2019) to generate molecular networking, and the data was visualized in Cytoscape. The LC-MS/MS data of the BuOH extracts of *Massilia* sp. YMA4 and its mutant strains (MSV000086803) are publicly available via MassIVE (https://massive.ucsd.edu, accessed on 3 February 2021).

## 4. Conclusions

In summary, we explored the cyclic lipopeptide, empedopeptin, and its analogs from *Massilia* sp. YMA4 using an integrated omics approach. To elucidate the biosynthetic mechanism of empedopeptin, we constructed five empedopeptin biosynthetic gene null-mutants of *Massilia* sp. YMA4. The antibiotic activities of *Massilia* sp. YMA4 mutants were decreased in comparison with the wild-type, demonstrating that empedopeptins were the primary antibiotic substances of *Massilia* sp. YMA4. In the comparative metabolomics analysis of wild-type and null-mutant strains, we successfully identified 44 empedopeptin analogs, including 17 cyclic lipopeptides and 27 linear lipopeptides. Through MultiGeneBlast analysis, we were also able to survey the similar BGCs of empedopeptin from other microbes, including *D. sacchari*, three *Collimonas*, and three *Variovorax* strains. Our findings illustrated that this integrated omics strategy is suitable to identify and develop potential bioactive metabolites from microbial sources without further isolation and purification.

## Data Availability

Data is contained within the article and Supplementary Materials.

## Supplementary Materials

The following are available online at https://www.mdpi.com/article/10.3390/md19040209/s1, Figure S1: Phylogenetic tree of genus *Massilia*, Figure S2: Mass spectrum and fragments information of empedopeptin, Figure S3: Schematic diagram of insertion mutant for *Massilia* sp. YMA4, Figure S4: PCR check of mutants. Table S1: Secondary metabolite gene clusters identified in *Massilia* sp. YMA4, Table S2: Structure information of cyclic lipopeptides, Table S3: Structure information of linear lipopeptides, Table S4: Genetic context and features of the BGC 6 in *Massilia* sp. YMA4 and its related BGCs in various microbes, Table S5: Oligonucleotides primer sets used in this study, Table S6: Primer sets for empedopeptin biosynthetic genes of *Massilia* sp. YMA4.
