# Supplementary material for: Integrated Omics Strategy Reveals Cyclic Lipopeptides Empedopeptins from Massilia sp. YMA4 and Their Biosynthetic Pathway
Source: Mar Drugs. 2021 Apr 9;19(4):209. doi: 10.3390/md19040209 (PMC8069584; doi:10.3390/md19040209)
Supplement: Supplementary file 1 [file marinedrugs-19-00209-s001.pdf]

Supplementary Information

Integrated Omics Strategy Reveals Cyclic Lipopeptides Empedopeptins from *Massilia* sp. YMA4 and their Biosynthetic Pathway

Shang-Tse Ho[1, 2#], Ying-Ning Ho[3, 4#], Chih Lin[1], Wei-Chen Hsu[1], Han-Jung Lee[1], Chia-Chi Peng[1], Han-Tan Cheng[1], Yu-Liang Yang[1, *]

[1] Agricultural Biotechnology Research Center, Academia Sinica, Taipei 11529, Taiwan

[2] Department of Wood Based Materials and Design, National Chiayi University, Chiayi 60004, Taiwan

[3] Institute of Marine Biology and Center of Excellence for the Oceans, National Taiwan Ocean University, Keelung 20224, Taiwan

[4] Center of Excellence for the Oceans, National Taiwan Ocean University, Keelung 20224, Taiwan

Tables

Table S1. Secondary metabolite gene clusters identified in *Massilia* sp. YMA4 2
Table S2. Structure information of cyclic lipopeptides 3
Table S3. Structure information of linear lipopeptides 4
Table S4. Genetic context and features of the BGC 6 in *Massilia* sp. YMA4 and its related BGCs in various microbes. 6
Table S5. Oligonucleotides primer sets used in this study 9
Table S6. Primer sets for empedopeptin biosynthetic genes of *Massilia* sp. YMA4 10

Figures

Figure S1. Phylogenetic tree of genus *Massilia*. 11
Figure S2. Tandem mass spectrum and fragment annotation of empedopeptin 12
Figure S3. Schematic diagram of insertion mutant for *Massilia* sp. YMA4 13
Figure S4. PCR check of mutants. 14

Table S1. Secondary metabolite gene clusters identified in *Massilia* sp. YMA4 by genome mining. Identified secondary metabolite clusters were predicted by antiSMASH 5.1.

Clusters	Type	Most similar known cluster	Similarity[a]
1	Siderophore	Unknown	
2	Hserlactone	Obafluorin	14%
3	Terpene	Unknown	
4	Acyl amino acids	Unknown	
5	NRPS	Turnerbactin	76%
6	NRPS	Entolysin	8%
7	Bacteriocin	Unknown	
8	NRPS, arylpolyene	APE Cf	10%
9	Thiopeptides	Unknown	
10	Hserlactone	Unknown	
11	Terpene	Carotenoid	100%
12	NRPS	Unknown	

[a] The percentage of genes within the closest known compound that have a significant BLAST hit to genes within the current gene cluster.

Table S2. Structure information of cyclic lipopeptides (core structure: 3-OH-Fatty acid-AA1-AA2-AA3-AA4-AA5-AA6-AA7-AA8)

Producing strains	Formula	$[M+H]^+$	$[M+H]^+$ calcd[a]	Error[b]	Sequences (AA8 to AA1)	3-OH-fatty acid
Δ*empB*	$C_{49}H_{79}N_{11}O_{16}$	1078.5750	1078.5785	-3.2	D/P/S/D/R/P/S/P	C14
wt[c]	$C_{47}H_{75}N_{11}O_{18}$	1082.5368	1082.5370	-0.2	3-OH-D/P/S/3-OH-D/R/P/S/P	C12
wt	$C_{46}H_{73}N_{11}O_{19}$	1084.5156	1084.5162	-0.6	3-OH-D/3-OH-P/S/3-OH-D/R/P/S/P	C11
Δ*empA*	$C_{49}H_{79}N_{11}O_{17}$	1094.5679	1094.5734	-5.0	D/P/S/3-OH-D/R/P/S/P	C14
Δ*empB*	$C_{49}H_{79}N_{11}O_{17}$	1094.5699	1094.5734	-3.2	3-OH-D/P/S/D/R/P/S/P	C14
wt	$C_{47}H_{75}N_{11}O_{19}$	1098.5315	1098.5319	-0.4	3-OH-D/3-OH-P/S/3-OH-D/R/P/S/P	C12
Δ*empB*	$C_{51}H_{81}N_{11}O_{16}$	1104.5927	1104.5941	-1.3	D/P/S/D/R/P/S/P	C16:1
Δ*empB*	$C_{51}H_{83}N_{11}O_{16}$	1106.6094	1106.6098	-0.4	D/P/S/D/R/P/S/P	C16
wt	$C_{48}H_{77}N_{11}O_{19}$	1112.5481	1112.5475	0.5	3-OH-D/3-OH-P/S/3-OH-D/R/P/S/P	C13
Δ*empB*	$C_{49}H_{79}N_{11}O_{17}$	1120.5876	1120.5890	-1.2	3-OH-D/P/S/D/R/P/S/P	C16:1
Δ*empA*	$C_{51}H_{83}N_{11}O_{17}$	1122.6049	1122.6047	0.2	D/P/S/3-OH-D/R/P/S/P	C16
Δ*empB*	$C_{51}H_{83}N_{11}O_{17}$	1122.6036	1122.6047	-1.0	3-OH-D/P/S/D/R/P/S/P	C16
all strains	$C_{49}H_{79}N_{11}O_{19}$	1126.5630	1126.5632	-0.2	3-OH-D/3-OH-P/S/3-OH-D/R/P/S/P	C14
Δ*empB*	$C_{51}H_{81}N_{11}O_{18}$	1136.5819	1136.5839	-1.8	3-OH-D/3-OH-P/S/D/R/P/S/P	C16
wt	$C_{50}H_{81}N_{11}O_{19}$	1140.5783	1140.5788	-0.4	3-OH-D/3-OH-P/S/3-OH-D/R/P/S/P	C15
wt	$C_{51}H_{81}N_{11}O_{19}$	1152.5775	1152.5788	-1.1	3-OH-D/3-OH-P/S/3-OH-D/R/P/S/P	C16:1
wt	$C_{51}H_{83}N_{11}O_{19}$	1154.5932	1154.5945	-1.1	3-OH-D/3-OH-P/S/3-OH-D/R/P/S/P	C16

[a] Calculated $[M + H]^+$, [b] ppm, [C] Wild-type.

Table S3. Structure information of linear lipopeptides (core structure: 3-OH-Fatty acid-AA1-AA2-AA3-AA4-AA5-AA6-AA7-AA8)

Producing strains	Formula	$[M+H]^+$	$[M+H]^+$ calcd[a]	Error[b]	Sequences	3-OH-fatty acid
	**6 AAs**				**AA6 to AA1**	
wt[c], Δ*empA*	$C_{38}H_{65}N_9O_{14}$	872.4707	872.4729	-2.5	S/3-OH-D/R/P/S/P	C12
wt, Δ*empA*	$C_{40}H_{69}N_9O_{14}$	900.5041	900.5042	-0.1	S/3-OH-D/R/P/S/P	C14
Δ*empA*	$C_{41}H_{71}N_9O_{14}$	914.5175	914.5199	-2.6	S/3-OH-D/R/P/S/P	C15
wt, Δ*empA*	$C_{40}H_{69}N_9O_{15}$	916.4994	916.4991	0.3	S/3-OH-D/R/3-OH-P/S/P	C14
wt, Δ*empA*	$C_{42}H_{71}N_9O_{14}$	926.5195	926.5199	-0.4	S/3-OH-D/R/P/S/P	C16:1
wt, Δ*empA*	$C_{42}H_{73}N_9O_{14}$	928.5344	928.5355	-1.2	S/3-OH-D/R/P/S/P	C16
	**7 AAs**				**AA7 to AA1**	
wt, Δ*empA*	$C_{43}H_{72}N_{10}O_{15}$	969.5242	969.5257	-1.5	P/S/3-OH-D/R/P/S/P	C12
Δ*empB*	$C_{45}H_{76}N_{10}O_{14}$	981.5599	981.5621	-2.2	P/S/D/R/P/S/P	C14
wt, Δ*empA*	$C_{43}H_{72}N_{10}O_{16}$	985.5182	985.5206	-2.4	3-OH-P/S/3-OH-D/R/P/S/P	C12
Δ*empB*	$C_{46}H_{78}N_{10}O_{14}$	995.5744	995.5777	-3.3	P/S/D/R/P/S/P	C15
wt, Δ*empA*	$C_{45}H_{76}N_{10}O_{15}$	997.5558	997.5570	-1.2	P/S/3-OH-D/R/P/S/P	C14
Δ*empB*	$C_{47}H_{78}N_{10}O_{14}$	1007.5753	1007.5777	-2.4	P/S/D/R/P/S/P	C16:1
Δ*empB*	$C_{47}H_{80}N_{10}O_{14}$	1009.5840	1009.5834	0.6	P/S/D/R/P/S/P	C16
Δ*empA*	$C_{46}H_{78}N_{10}O_{15}$	1011.5728	1011.5726	0.2	P/S/3-OH-D/R/P/S/P	C15
wt, Δ*empA*	$C_{45}H_{76}N_{10}O_{16}$	1013.5517	1013.5519	-0.2	3-OH-P/S/3-OH-D/R/P/S/P	C14
wt, Δ*empA*	$C_{47}H_{78}N_{10}O_{15}$	1023.5724	1023.5726	-0.2	P/S/3-OH-D/R/P/S/P	C16:1
wt, Δ*empA*	$C_{47}H_{80}N_{10}O_{15}$	1025.5853	1025.5883	-2.9	P/S/3-OH-D/R/P/S/P	C16
wt, Δ*empA*	$C_{45}H_{76}N_{10}O_{17}$	1029.5483	1029.5468	1.5	3-OH-P/S/3-OH-D/R/3-OH-P/S/P	C14
wt, Δ*empA*	$C_{47}H_{78}N_{10}O_{16}$	1039.5680	1039.5679	0.1	3-OH-P/S/3-OH-D/R/P/S/P	C16:1
Δ*empA*	$C_{48}H_{82}N_{10}O_{15}$	1039.6040	1039.6041	-0.1	P/S/3-OH-D/R/P/S/P	C17

	8 AAs				AA8 to AA1	
wt	$C_{47}H_{77}N_{11}O_{20}$	1114.5269	1114.5268	0.1	3-OH-D/3-OH-P/S/3-OH-D/R/P/S/P	C12:1
wt	$C_{47}H_{77}N_{11}O_{20}$	1116.5414	1116.5425	-1.0	3-OH-D/3-OH-P/S/3-OH-D/R/P/S/P	C12
wt	$C_{49}H_{81}N_{11}O_{20}$	1144.5723	1144.5738	-1.3	3-OH-D/3-OH-P/S/3-OH-D/R/P/S/P	C14
wt	$C_{51}H_{83}N_{11}O_{20}$	1170.5879	1170.5894	-1.3	3-OH-D/3-OH-P/S/3-OH-D/R/P/S/P	C16:1
wt	$C_{51}H_{85}N_{11}O_{20}$	1172.6031	1172.6051	-1.7	3-OH-D/3-OH-P/S/3-OH-D/R/P/S/P	C16
wt	$C_{51}H_{85}N_{11}O_{21}$	1188.6003	1188.6000	0.3	3-OH-D/3-OH-P/S/3-OH-D/R/P/S/P	C12
wt	$C_{53}H_{87}N_{11}O_{20}$	1198.6215	1198.6207	0.7	3-OH-D/3-OH-P/S/3-OH-D/R/P/S/P	C18:1

[a] Calculated $[M + H]^+$, [b] ppm, [c] Wild-type.

Table S4. Genetic context and features of the BGC 6 in *Massilia* sp. YMA4 and its related BGCs in various microbes

Microbes	Accession number	Gene name (Locus tag)	Proposed function	AA length
Massilia sp. YMA4	CP030092.1	*empA* (DPH57_09130)	TauD/TfdA family dioxygenase	305
		empB (DPH57_09135)	TauD/TfdA family dioxygenase	326
		empC (DPH57_09140)	non-ribosomal peptide synthetase	2751
		empD (DPH57_09145)	amino acid adenylation domain-containing protein	1122
		empE (DPH57_09150)	amino acid adenylation domain-containing protein	5322
C. fungivorans ESAIA1	RAIL01000001.1	*geneA* (DFH22_2667)	TfdA family taurine catabolism dioxygenase TauD	308
		geneB (DFH22_2666)	TauD/TfdA family dioxygenase	317
		geneC (DFH22_2665)	amino acid adenylation domain-containing protein	2756
		geneD (DFH22_2664)	amino acid adenylation domain-containing protein	1171
		geneE (DFH22_2663)	amino acid adenylation domain-containing protein	5358
C. fungivorans Ter331	CP002745.1	*geneA* (CFU_2180)	TauD/TfdA family dioxygenase	274
		geneB (CFU_2181)	TauD/TfdA family dioxygenase	317
		geneC (CFU_2182)	putative non-ribosomal peptide synthetase	2815
		geneD (CFU_2184)	non-ribosomal peptide synthetase	1168
		geneE (CFU_2185)	amino acid adenylation	5375
C. fungivorans Ter6	CP013232.1	*geneA* (CFter6_2734)	TauD/TfdA family dioxygenase	305
		geneB (CFter6_2735)	TauD/TfdA family dioxygenase	317
		geneC (CFter6_2736)	amino acid adenylation domain-containing protein	2763
		geneD (CFter6_2737)	non-ribosomal peptide synthetase	1170
		geneE (CFter6_2738)	non-ribosomal peptide synthetase	5361

Duganella sacchari	FRCX01000009.1	*geneA* (SAMN05192549_102249)	TauD/TfdA family dioxygenase	305
		geneB (SAMN05192549_102250)	TauD/TfdA family dioxygenase	316
		geneC (SAMN05192549_102251)	non-ribosomal peptide synthetase	2747
		geneD (SAMN05192549_102252)	AMP-binding protein	871
		geneE (SAMN05192549_102253)	non-ribosomal peptide synthetase	5334
V. guangxiensis	RXFT01000001.1	*geneA* (EJP67_06565)	TauD/TfdA family dioxygenase	304
		geneB (EJP67_06570)	TauD/TfdA family dioxygenase	328
		geneC (EJP67_06575)	amino acid adenylation domain-containing protein	2794
		geneD (EJP67_06580)	amino acid adenylation domain-containing protein	1172
		geneE (EJP67_06585)	amino acid adenylation domain-containing protein	5529
Variovorax sp. OV084	FOII01000021.1	*geneA* (SAMN05443580_1317)	TauD/TfdA family dioxygenase	318
		geneB (SAMN05443580_1316)	Taurine dioxygenase, alpha-ketoglutarate-dependent	314
		geneC (SAMN05443580_1315)	amino acid adenylation domain-containing protein	2767
		geneD (SAMN05443580_1314)	non-ribosomal peptide synthetase	1166
		geneE (SAMN05443580_1313)	non-ribosomal peptide synthetase	5350

Variovorax sp. YR752	OCMW01000002.1	*geneA* (SAMN05518800_2816)	TauD/TfdA family dioxygenase	314
		geneB (SAMN05518800_2815)	Taurine dioxygenase, alpha-ketoglutarate-dependent	318
		geneC (SAMN05518800_2814)	amino acid adenylation domain-containing protein	2765
		geneD (SAMN05518800_2813)	non-ribosomal peptide synthetase	1170
		geneE (SAMN05518800_2812)	amino acid adenylation domain-containing protein	5355

[a] The proposed function of each gene was predicted by NCBI BlastP.

Table S5. Oligonucleotides primer sets used in this study

Primer	Sequence (5'->3')
**qRT-PCR**	
16S-F	CCTGAAGAATAAGCACCGGCTAACTA
16S-R	GGGGATTTCACGACAGACTTACAAAA
empA-F	CTTGTACAGGGTGTTGCACG
empA-R	ATCAACCATGAACGACGGGA
empB-F	GCCTCGATCAGGTAGGGGAA
empB-R	CACTACTGGACGAACACACCA
empC-F	TTGTTTGCGTCGAACAGCTC
empC-R	CCCAATGCGATACCGGAGAG
empD-F	CAGGTCCTCCTTGTGCGAA
empD-R	TTGAGGAGGTCGAGCTGTGA
empE-F	CAACGACGCCGATATTGCAG
empE-R	CCCCCTGCGTCTGTTTTCAA
**PCR**	
empA-ck1-F	ATCAAAGGTACGCAAGGCGA
empA-ck2-R	GATCGAGCAATCGGTCAGCA
empB-ck1-F	CTTTCGAGGTGACGGCCA
empB-ck2-R	TGTTCGACGCATGCCACA
empC-ck1-F	GACGCAAACAAGGTCGAGGT
empC-ck2-R	CTCTCGATGTGGCGGATACC
empD-ck1-F	GCACTGGAATTGCTGGACAA
empD-ck2-R	CGTGTTGAGGAACATGCCCA
empE-ck1-F	TCAGTTGAGTTCACCCCAGC
empE-ck2-R	GTAGCAGCGTTTCAGTTCCG
pCM184-ck3-R	CGAACGACATGGAGCGGCAC
pCM184-ck4-2-F	AAAGCGGTTAGCTCCTTCGG

Table S6. Primer sets for empedopeptin biosynthetic genes of *Massilia* sp. YMA4

Target gene	Primer set for positive PCR (left arm; primer 1 and 3)	Primer set for positive PCR (right arm; primer 2 and 4)	Primer set for negative PCR (primer 1 and 2)	PCR product size for negative PCR (wild type)	PCR product size for positive PCR (left arm)	PCR product size for positive PCR (right arm)
empA	*empA*-ck1-F/pCM184-ck3-R	pCM184-ck4-2-F/*empA*-ck2-R	*empA*-ck1-F/*empA*-ck2-R	780 bp	769 bp	1497 bp
empB	*empB*-ck1-F/pCM184-ck3-R	pCM184-ck4-2-F/*empB*-ck2-R	*empB*-ck1-F/*empB*-ck2-R	690 bp	905 bp	1477 bp
empC	*empC*-ck1-F/pCM184-ck3-R	pCM184-ck4-2-F/*empC*-ck2-R	*empC*-ck1-F/*empC*-ck2-R	1355 bp	972 bp	1612 bp
empD	*empD*-ck1-F/pCM184-ck3-R	pCM184-ck4-2-F/*empD*-ck2-R	*empD*-ck1-F/*empD*-ck2-R	1161 bp	644 bp	1280 bp
empE	*empE*-ck1-F/pCM184-ck3-R	pCM184-ck4-2-F/*empE*-ck2-R	*empE*-ck1-F/*empE*-ck2-R	949 bp	699 bp	1358 bp

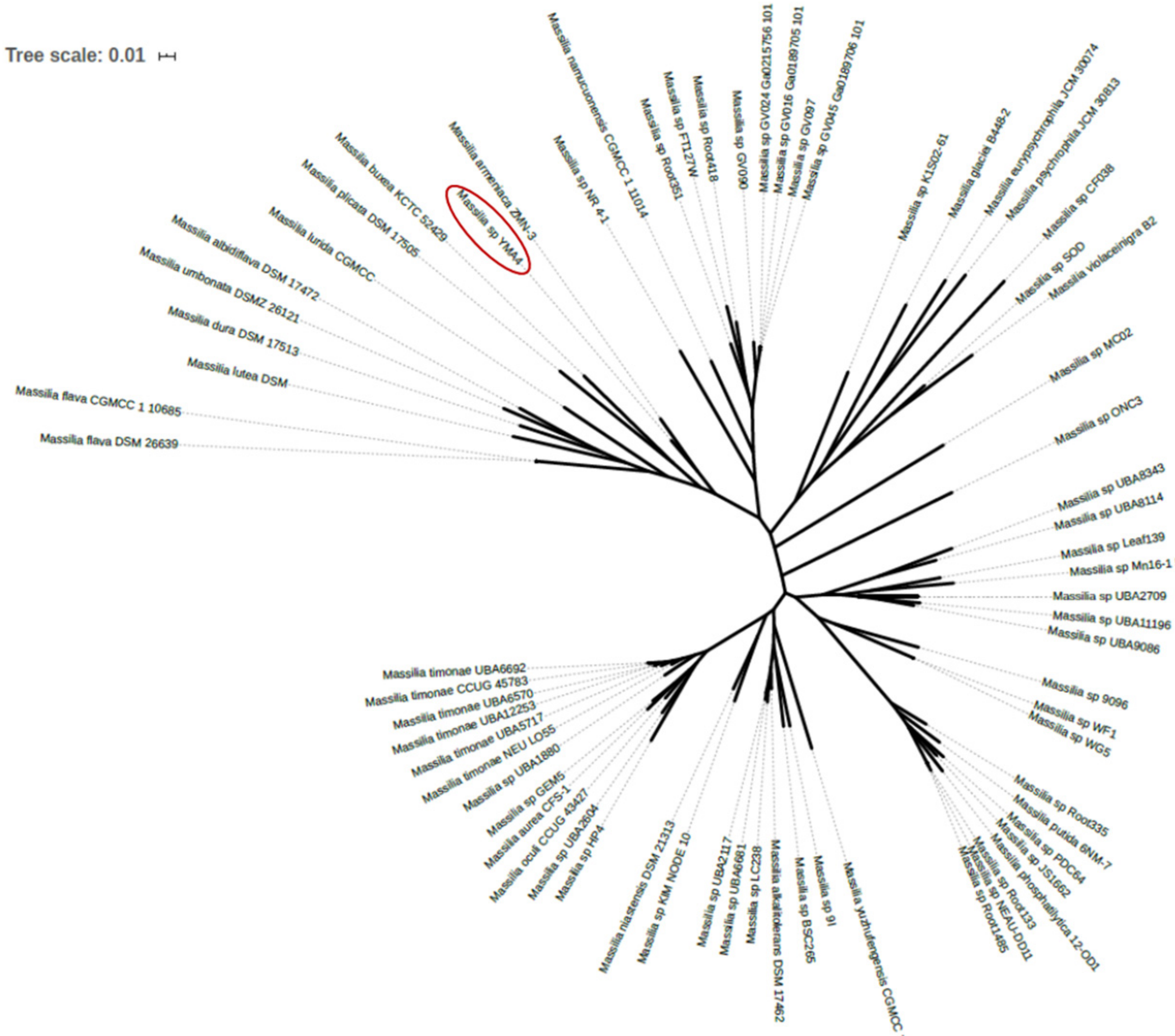

Figure S1. Phylogenetic tree of genus *Massilia*. The genome sequences of genus *Massilia* were downloaded from NCBI.

MS^2-1126.56-empedopeptin

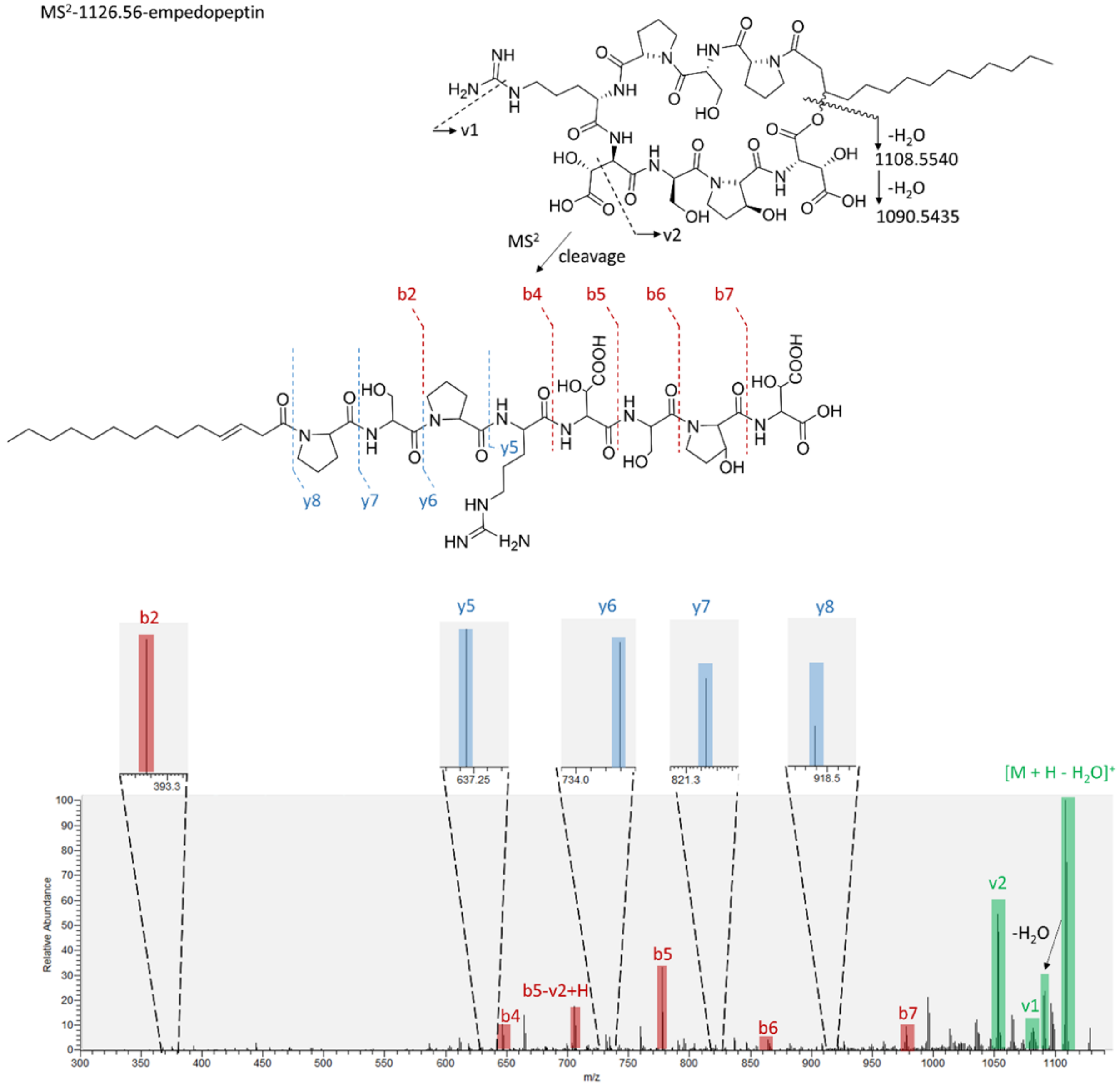

Observed mass [Da]	Calculated mass [Da]	Error (ppm)	Species
375.2656	375.2648	-2.1	b2 - H_2O
393.2738	393.2753	3.8	b2
411.2854	411.2859	1.2	b2 + H_2O
472.3171	472.3175	0.8	b3 - H_2O
637.2446	637.2429	-2.7	y5
646.4296	646.4292	-0.6	b4
664.4406	664.4398	-1.2	b4 + H_2O
705.4670	705.4660	-0.9	b5 - v2 + H
734.2978	734.2957	-2.9	y6
759.4416	759.4405	-1.4	b5 - H_2O
777.4520	777.4511	-1.2	b5
795.4645	795.4616	-3.6	b5 + H_2O
821.3257	821.3277	2.4	y7
846.4752	846.4725	-3.2	b6 - H_2O
864.4842	864.4831	-1.3	b6
882.4943	882.4937	-0.7	b6 + H_2O
918.3830	918.3805	-2.7	y8
959.5206	959.5202	-0.4	b7 - H_2O
977.5298	977.5308	1.0	B7
995.5426	995.5413	-1.3	b7 + H_2O
1052.5641	1052.5628	-1.2	v2
1084.5424	1084.5414	-0.9	v1
1090.5435	1090.5421	-1.3	$[M + H - 2H_2O]^+$
1108.5540	1108.5526	-1.3	$[M + H - H_2O]^+$

Figure S2. Tandem mass spectrum and fragment annotation of empedopeptin.

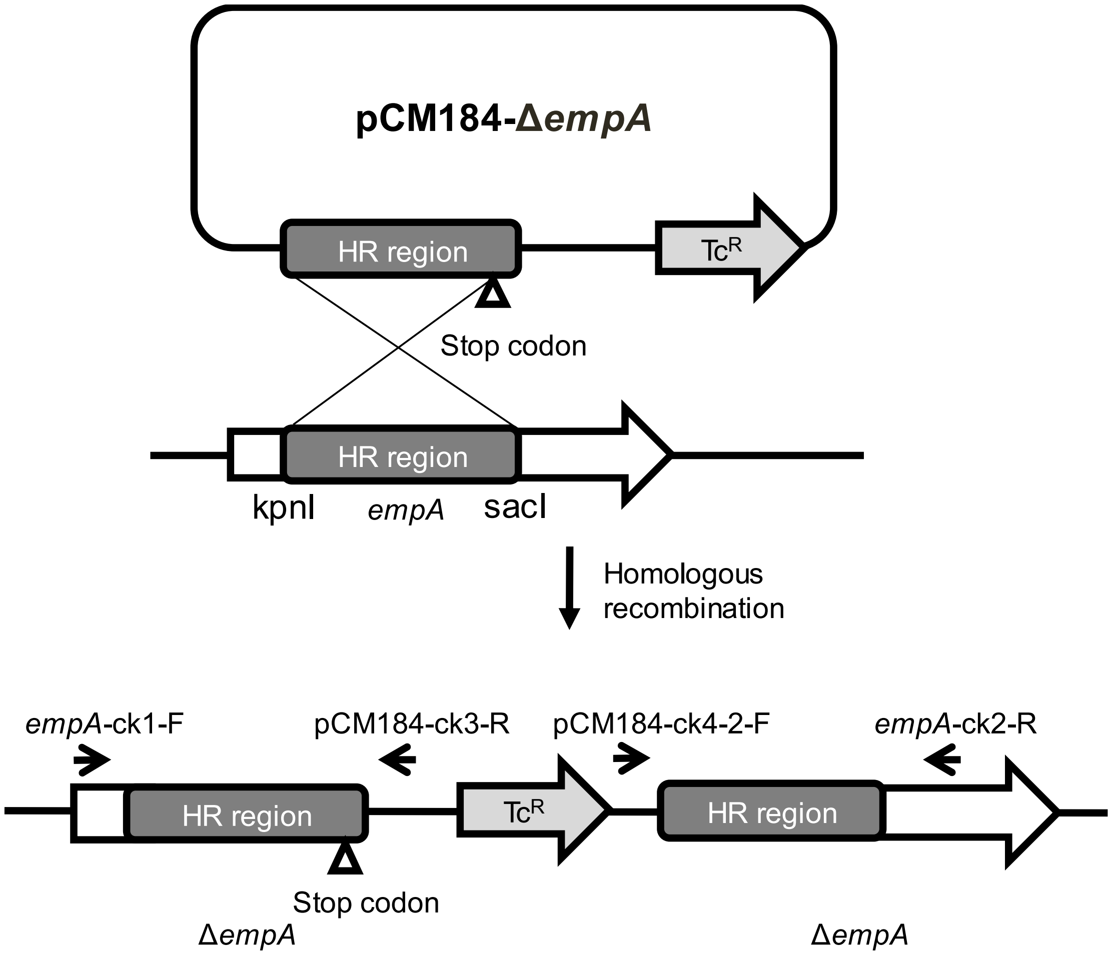

Figure S3. Schematic diagram of insertion mutant for *Massilia* sp. YMA4.

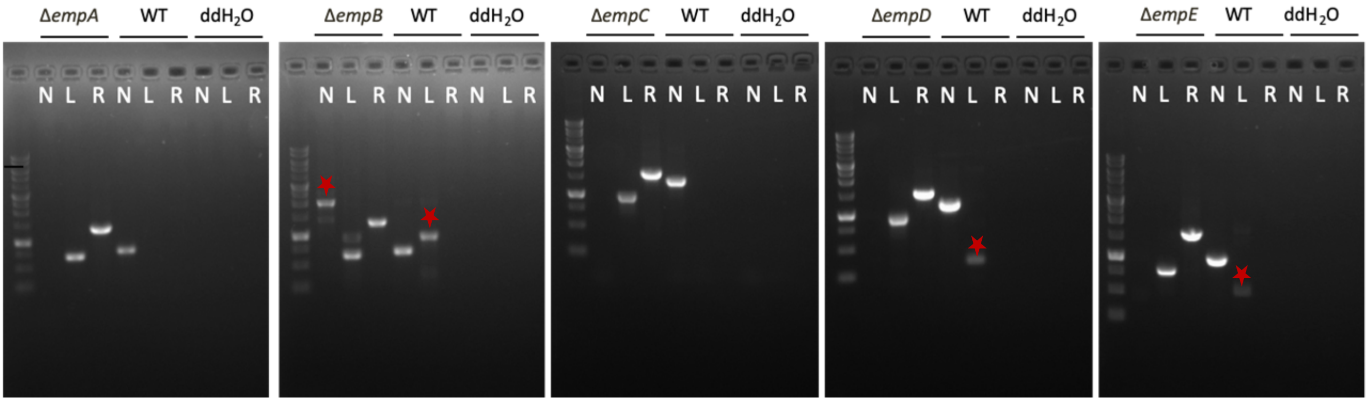

Figure S4. PCR check of mutants. The represented primer set of PCR check for Δ*empA*: N: *empA*-ck1-F + *empA*-ck2-R; L: *empA*-ck1-F + pCM184-ck3-R; R: pCM184-ck4-2-F + *empA*-ck2-R. The positions of primers were shown in **Figure S3**. The sequences of all the bands were confirmed and ✶ represented non-specific bands.
